# Effects of ketone body 3-hydroxybutyrate on cardiac and mitochondrial function during donation after circulatory death heart transplantation

**DOI:** 10.1038/s41598-024-51387-y

**Published:** 2024-01-08

**Authors:** Jacob Marthinsen Seefeldt, Yaara Libai, Katrine Berg, Nichlas Riise Jespersen, Thomas Ravn Lassen, Frederik Flyvholm Dalsgaard, Pia Ryhammer, Michael Pedersen, Lars Bo Ilkjaer, Michiel A. Hu, Michiel E. Erasmus, Roni R. Nielsen, Hans Erik Bøtker, Oren Caspi, Hans Eiskjær, Niels Moeslund

**Affiliations:** 1 Department of Cardiology, Aarhus University Hospital, Palle Juul-Jensens Boulevard 99, 8200 Aarhus N, Denmark; 2 Department of Clinical Medicine, Aarhus University, Palle Juul-Jensens Boulevard 82, 8200 Aarhus N, Denmark; 3 The Laboratory for Cardiovascular Precision Medicine, Rapport Faculty of Medicine, Technion and Rambam’s Cardiovascular Research and Innovation Center, 2 Efron St, Haifa, Israel; 4 Comparative Medicine Lab, Department of Clinical Medicine, Aarhus University, Palle Juul-Jensens Boulevard 82, 8200 Aarhus N, Denmark; 5 Department of Anesthesiology, Regional Hospital Silkeborg, Falkevej 1A, 8600 Silkeborg, Denmark; 6 Department of Cardiothoracic and Vascular Surgery, Aarhus University Hospital, Palle Juul-Jensens, Boulevard 99, 8200 Aarhus N, Denmark; 7 Department of Cardiothoracic Surgery, University Medical Center Groningen, Hanzeplein 1, 9713 GZ Groningen, The Netherlands

**Keywords:** Experimental models of disease, Preclinical research, Translational research, Cardiology, Heart failure

## Abstract

Normothermic regional perfusion (NRP) allows assessment of therapeutic interventions prior to donation after circulatory death transplantation. Sodium-3-hydroxybutyrate (3-OHB) increases cardiac output in heart failure patients and diminishes ischemia–reperfusion injury, presumably by improving mitochondrial metabolism. We investigated effects of 3-OHB on cardiac and mitochondrial function in transplanted hearts and in cardiac organoids. Donor pigs (n = 14) underwent circulatory death followed by NRP. Following static cold storage, hearts were transplanted into recipient pigs. 3-OHB or Ringer’s acetate infusions were initiated during NRP and after transplantation. We evaluated hemodynamics and mitochondrial function. 3-OHB mediated effects on contractility, relaxation, calcium, and conduction were tested in cardiac organoids from human pluripotent stem cells. Following NRP, 3-OHB increased cardiac output (P < 0.0001) by increasing stroke volume (P = 0.006), dP/dt (P = 0.02) and reducing arterial elastance (P = 0.02). Following transplantation, infusion of 3-OHB maintained mitochondrial respiration (P = 0.009) but caused inotropy-resistant vasoplegia that prevented weaning. In cardiac organoids, 3-OHB increased contraction amplitude (P = 0.002) and shortened contraction duration (P = 0.013) without affecting calcium handling or conduction velocity. 3-OHB had beneficial cardiac effects and may have a potential to secure cardiac function during heart transplantation. Further studies are needed to optimize administration practice in donors and recipients and to validate the effect on mitochondrial function.

## Introduction

Donation following circulatory death (DCD) is emerging as an alternative practice to donation after brain death due to a potential increase in donor numbers for heart transplantation^1,2^. In contrast to donation after brain death, which currently accounts for most solid organ transplantations today, DCD requires cessation of circulation and respiration, hence introducing warm ischemic injury prior to organ preservation^3,4^. Several preservation strategies are used after circulatory death. Normothermic regional perfusion (NRP) is increasingly used in Europe and the USA^4–6^. The combination of DCD and NRP is a novel strategy in human heart transplantation^5–7^. When blood flow is re-established by NRP, reperfusion might precondition the heart and restore depleted energy metabolites such as ATP before transplantation^8^. The use of NRP allows assessment of cardiac function before transplantation and provides an opportunity to begin preservative interventions that may reduce ischaemia‒reperfusion (IR) injury and improve posttransplantation outcomes^4^. Experimental studies have revealed that modulation of substrate supply by exogenous administration of the ketone body sodium-3-hydroxybutyric acid (3-OHB) seems to provide cardioprotective effects by improving functional recovery after myocardial infarction^9–12^. Moreover, infusion with 3-OHB in patients with heart failure (HF) increases cardiac output (CO) by 40% without compromising myocardial energy efficiency^13^. Nonetheless, it is unknown whether 3-OHB improves myocardial function following cardiac transplantation. During metabolic stress, such as HF or IR, ketones may modulate myocardial mitochondrial function^9,14^. While the hemodynamic effects of 3-OHB involve increased contractility and vasodilatation^15^, the mechanism for these isolated actions remains unknown. The aims of this study were to investigate whether 3-OHB infusion during NRP improves cardiac hemodynamic and mitochondrial function after cardiac transplantation and to investigate potential mechanisms by which 3-OHB affects the cardiovascular system.

## Materials and methods

The experimental protocol conformed to the Danish law for animal research and the guidelines from Directive 2010/63/EU of the European Parliament on the protection of animals used for scientific purposes. We followed the ARRIVE 2.0 guidelines^16,17^. The in vivo study was approved by the Danish National Committee on Animal Research Ethics with the protocol permission (2018-15-0201-01603) and conducted in accordance with the Principles of Laboratory Animal Care^18^. The in vitro study was approved by the Rambam Health Care Campus Helsinki Committee. Human induced pluripotent stem cells (HiPSC) were generated from healthy volunteers following informed consent and after institutional review board approval in accordance with the Helsinki Declaration^19^.

A more detailed description of the materials and methods is provided in the supplementary information.

### In vivo study design

**Figure 1:**
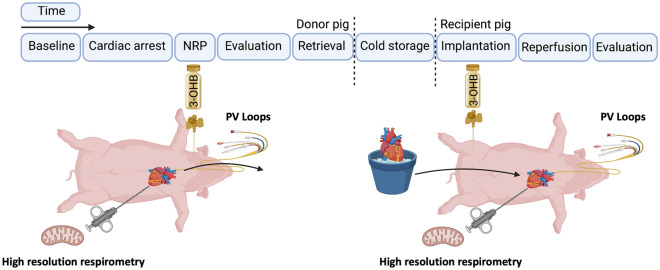
Schematic illustration of the experimental protocol. Created with Biorender.com.

We performed orthotopic heart transplantations from DCD donors followed by NRP and static cold storage (SCS) before transplantation on female Danish Landrace pigs (~ 80 kg). Fourteen donor pigs underwent circulatory death followed by NRP. Infusion of 3-OHB (n = 5) or Ringer’s acetate (n = 9) was initiated at the onset of NRP and until procurement in donors and reinitiated following implantation in recipient pigs with continuous administration until the end of the protocol (Fig. 1). Pigs were euthanized at the end of the protocol with 0.25 mL/kg pentobarbital (Euthanimal 400 mg/mL) administered intravenously. The Ringer’s acetate group was powered to serve as a DCD donor heart control cohort for other studies, whereas the 3-OHB group was exploratory^20^. Therefore, the initial number of pigs included differed between groups.

All analyses were conducted blinded to the interventions. The primary endpoint was the change in CO following NRP. Secondary endpoints included left ventricular functional parameters following NRP, assessed by pressure‒volume analysis, and mitochondrial respiratory capacity following transplantation, evaluated by high-resolution respirometry.

### Anaesthesia, monitoring, and baseline measurements

Upon arrival, the animals were orally intubated and mechanically ventilated. Anaesthesia and analgesia were maintained with inhaled sevoflurane (3%) and fentanyl (15 μg/kg/h). A Swan-Ganz (7.5F CCOmbo, Edwards Lifescience, USA) catheter was inserted through the right external jugular vein. A pressure‒volume admittance catheter, 5Fr (Transonic Systems, Ithaca, USA), was inserted into the left ventricle.

### Donor procedure

A median sternotomy was performed, and heparin (40,000 IU) was administered to achieve systemic anticoagulation. The ascending aorta and right atrium were cannulated and connected to the NRP circuit, which comprised a standard roller pump cardiopulmonary bypass (CPB). After instrumentation, baseline parameters were recorded, and blood and tissue were sampled.

### DCD, NRP and intervention

Following baseline recording, mechanical ventilation was disconnected, resulting in asphyxiation and circulatory arrest. An additional 10 min of warm ischemia was added after circulatory arrest to uphold a “5 min no touch” and mimic the preparation time for NRP in a clinical setting. After the 10-min circulatory arrest period, NRP and ventilation were initiated and maintained for 60 min. Hemodynamic support with dobutamine (2.5 µg/kg/min) and norepinephrine titrated to MAP > 60 mmHg was used during NRP. Treatment with 3-OHB (0,360 mg/kg/h equivalent to 4.8 mL/kg/h) or isotonic Ringer’s acetate (control) commenced at the onset of NRP and continued until donor heart procurement. After the 60-min NRP period, the animal was fully weaned and observed for 30 min followed by acquisition of hemodynamic parameters, blood, and tissue samples. Procurement was initiated with organ flush and storage with Custodiol® Histidine-tryptophan-ketoglutarate solution at 4 °C.

### Recipient procedure

Animal preparation, monitorization and thoracic exposure were performed as in the donor protocol. Systemic anticoagulation with 40,000 IU heparin and ascending aortic and bicaval cannulation were used to institute CPB before orthotopic heart transplantation using the bicaval technique was performed^20^. The transplanted hearts were reperfused and left unloaded for 60 min before weaning attempts commenced.

### Hemodynamic evaluation by pressure‒volume catheters

We recorded cardiac output (CO), stroke volume (SV), arterial elastance (Ea), first derivatives of pressure (dP/dt max), end systolic pressure (ESP), end diastolic pressure (EDP), end systolic volume (ESV), end diastolic volume (EDV), end systolic elastance (Ees) and stroke work (SW).

### Mitochondrial respiratory capacity

We analysed mitochondrial respiratory capacity using a high-resolution respirometer (Oxygraph-2K; Oroboros Instruments, Innsbruck, Austria), as previously described^21–24^. Biopsies were taken from the anterior apical superficial myocardium of the LV using a bioptome directly through the open thorax to secure the location of the left ventricle and to leave the PV system undisturbed.

Saponin-permeabilized fiber bundles were added to the chambers in the Oxygraph-2k chambers, and physiological mitochondrial respiration focused on complexes I and II was evaluated.

The respiratory rates are expressed as the O_2_ flux and normalized to the cardiac muscle wet weight of the permeabilized fibers. We evaluated the net capacity for mitochondrial oxidative phosphorylation for complexes I and II in the presence of ADP (OXPHOS capacity) and the respiratory control ratio (RCR)^25^.

### In vitro hiPSC study

Dermal fibroblasts were obtained from healthy individuals. Fibroblasts were reprogrammed to generate patient-specific HiPSCs^26,27^ and further differentiated into cardiomyocytes^28^. Cardiomyocyte differentiation was previously confirmed with immunostaining using nuclear staining marker DAPI and CY2 for cardiac troponin (Fig. 4)^29^.

### Microorganoid tissue engineering

HiPSCs derived cardiomyocytes were incubated for 7 days before video recordings were carried out using a robotic inverted microscope (Olympus IX83; Olympus, USA). A 96-well plate was randomly divided into two groups containing 48 wells each: 10 mM NaCl (control) or 10 mM DL-3-OHB (3-OHB) (Acros Organics) was administered depending on the groups 20 min before recording. The concentrations used were based on previous dose–response experiments in our group^15^. The videos were analysed using the MUSCLEMOTION tool for ImageJ (https://imagej.net/ij/index.html)^30,31^.

### Calcium imaging

HiPSC-derived cardiomyocytes were loaded with the calcium indicator Fluo-4 AM (Thermo Fisher, USA), and calcium fluorescent signals were recorded with a confocal imaging system (Zeiss LSM900 with Airyscan 2, Germany) in clusters of 2–3 cells. The data were analysed using a custom written MATLAB program (MathWorks, USA) as previously described^32^. Four wells of each group control and 3-OHB were included in the analysis.

### Optical mapping

HiPS-derived cardiomyocytes were plated in a surface 35 mm culture dish (Corning CellBIND, USA) in a monolayer form and loaded with a FluoVolt Membrane Potential dye kit (D10488, Thermo Fisher, USA). All experiments were paced (1–2 Hz) using a bipolar electrode. Imaging was performed using a fast electron-multiplying charge-coupled device (EM-CCD) camera (Evolve® 512 Delta, Photometrics, 512 × 512 pixel) mounted on a fluorescence microscope (Olympus MVX10; Olympus, Japan).

### Statistics

Q‒Q plots were used to assess continuous data for normality. Normally distributed data are presented as the mean and standard deviation. Nonnormally distributed data are presented as the median and interquartile range. Comparisons of means between groups were analysed by a student’s t test, mixed effects model to allow for missing data of ANOVA for normally distributed data and Mann‒Whitney U test for nonnormally distributed data. Bonferroni’s multiple comparisons post hoc test was used for multiple comparisons in the in vivo studies. Levene’s test for equal variance was used for the in vitro* studies.*

All analyses were performed using GraphPad Prism 9.3.1 (Graph Pad Software, CA, USA). P < 0.05 was considered statistically significant.

## Results

### In vivo protocol feasibility

All fourteen DCD donor hearts were successfully reanimated using NRP, procured and implanted in recipient 3-OHB (n = 5) and control (n = 9) pigs. Ischemia times in donors and recipients were similar in the 3-OHB (n = 5) and control (n = 9) groups, functional warm ischemic time: (3-OHB vs. control (median (IQ range)): 13 (11.5–14.5) vs. 13 (11.5–17), P = 0.54) (Table S1). The vasoactive inotropic score^33^ was similar between 3-OHB (n = 5) and control (n = 9) groups after weaning from NRP: (3-OHB vs. control (median (IQ range)): 1.3 (0.68–4.7) vs. (2.5 (1.35–3.36), P = 0.90) (Table S1). However, recipient pigs treated with 3-OHB could not be weaned from CPB post transplantation due to norepinephrine-resistant vasoplegia; therefore, only hemodynamic comparisons in the donor pigs after NRP were performed, while mitochondrial respiratory capacity was evaluated both before and after transplantation.

### Hemodynamic effects of 3-OHB infusion in donor pigs

**Figure 2:**
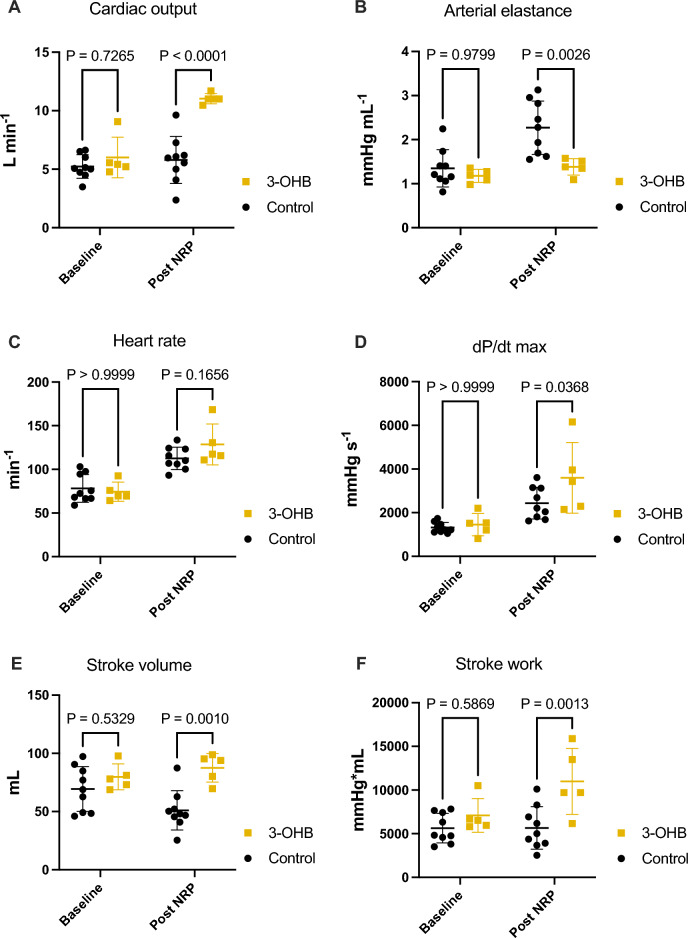
Hemodynamic and PV recordings. (**A**) Cardiac output. (**B**) Arterial elastance. (**C**) Heart rate. (**D**) dP/dt max. (**E**) Stroke volume. (**F**) Stroke work. Baseline after stabilization. Post NRP: 30 min after the end of NRP. *NRP* normothermic regional perfusion, *3-OHB* sodium-3-hydroxybutyric acid, *dP/dt max* derivative of pressure over time, *Ees* end systolic elastance. Data are mean with bars indicating standard deviation. P < 0.05 was considered significant.

Continuous infusion with 3-OHB (360 mg/kg/h) increased plasma levels of 3-OHB from 0.05 ± 0.06 mM to 3.95 ± 1.13 mM (P = 0.006) following NRP. This was associated with an increase in CO from 6.0 ± 1.7 L × min^−1^ to 11.0 ± 0.4 L × min^−1^ (P < 0.0001), with only a slight increase in controls from 5.2 ± 1.0 L × min^−1^ to 5.80 ± 2.0 L × min^−1^ (P = 0.4) (Fig. 2). Arterial elastance was simultaneously reduced in the 3-OHB group compared with the controls (1.4 ± 0.2 mmHg × min^−1^ vs. 2.3 ± 0.6 mmHg × min^−1^, P = 0.003) (Fig. 2). Whereas SV was similar between groups at baseline (P = 0.5), SV was higher in the 3-OHB group after NRP and 3-OHB infusion (P < 0.001) (Fig. 2). EF was preserved by 3-OHB but decreased in the control group compared with baseline (Table S3). Dp/dt max was increased by 3-OHB compared with the control (P = 0.04) (Fig. 2). This resulted in an increase in stroke work in the 3-OHB group compared with the control group (Fig. 2). HR and MAP increased after NRP compared with baseline in both groups (P < 0.05), but there were no differences between groups (P = 0.25) (Fig. 2 and Table S3).

ESV, EDV, ESP and EDP were all equal between groups at baseline and unchanged during the protocol, except for an increase in ESP in control pigs following NRP (Table S3).

### Mitochondrial respiration

**Figure 3:**
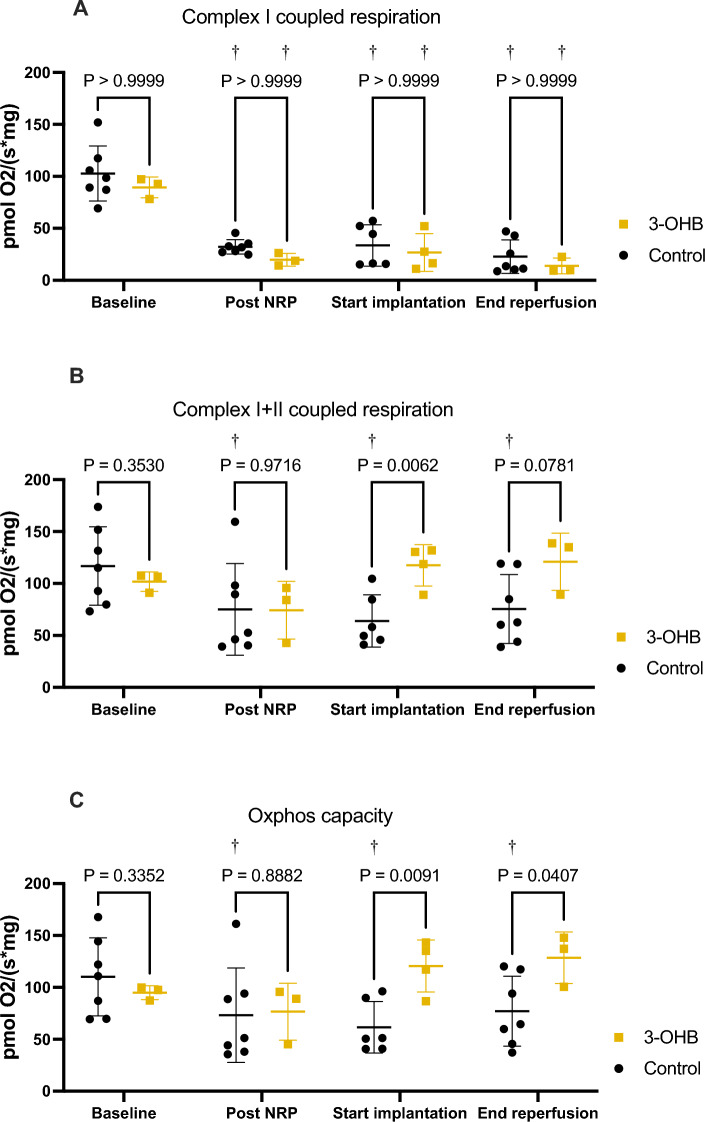
Mitochondrial respiratory capacity measured by high resolution respirometry. (**A**): Complex I respiration with glutamate, malate and ADP. (**B**) Complex I + II respiration with glutamate, malate, ADP and succinate. (**C**) Oxphos capacity: calculated as the absolute difference between complex I + II respiration and leak state respiration. Baseline: after stabilization. Post NRP: 60 min after start of NRP. Start implantation: after implantation but before reperfusion. End reperfusion: 60 min after the start of reperfusion. *3-OHB* sodium-3-hydroxybutyric acid, *Oxphos* oxidative phosphorylation. Data are mean with bars indicating standard deviation. ^†^P < 0.05 compared with baseline. P < 0.05 was considered significant.

Mitochondrial respiration specific to complex I (glutamate and malate) with ADP (state 3) declined significantly following NRP, implantation and reperfusion (Fig. 3). The decrease did not differ between the 3-OHB and control groups (Fig. 3). In contrast, OXPHOS capacity was enhanced after implantation in the 3-OHB group compared with the control (rmolO_2_ × s^−1^ × mg^−1^: 121 ± 25 vs. 62 ± 25, P = 0.009) and remained stable after reperfusion (rmolO_2_ × s^−1^ × mg^−1^:129 ± 25 vs. 77 ± 34, P = 0.04) (Fig. 3). Furthermore, a decline in OXPHOS capacity following transplantation was observed in the control group but not in the 3-OHB group (Fig. 3). Complexes I and II respiration combined and in the presence of ADP was enhanced in the 3-OHB group compared to control after implantation (rmolO_2_ × s^−1^ × mg^−1^: 141 ± 25 vs. 78 ± 26, P = 0.006) and at the end of reperfusion (rmolO_2_ × s^−1^ × mg^−1^: 128 ± 29 vs. 89 ± 33, P = 0.07) (Fig. 3).

3-OHB increased state 4o leak respiration, as assessed by titration of oligomycin, to a larger extent than the control after implantation (rmolO_2_ × s^−1^ × mg^−1^: 111 ± 26 vs. 57 ± 27, P = 0.02) and reperfusion (rmolO_2_ × s^−1^ × mg^-1^: 122 ± 19 vs. 70 ± 34, P = 0.02) (Fig. S2).

Finally, when comparing respiration with glucose-linked substrates and 3-OHB-linked substrates in the high-resolution respirometry chamber, cardiac transplantation caused a shift in substrate preference that was most pronounced in control pigs (RCR for 3-OHB vs. glucose-linked substrates at end reperfusion: 22.26 ± 12.37 vs. 8.06 ± 3.20, P = 0.0008) (Fig. S2B) but also apparent in 3-OHB pigs (RCR for 3-OHB vs. glucose-linked substrates at end reperfusion: 38.45 ± 30.34 vs. 14.23 ± 1.22) (Fig. S2C).

### 3-OHB effect on cardiac micro-organoid tissue

No variable differed between groups at baseline (Fig. S3).

**Figure 4:**
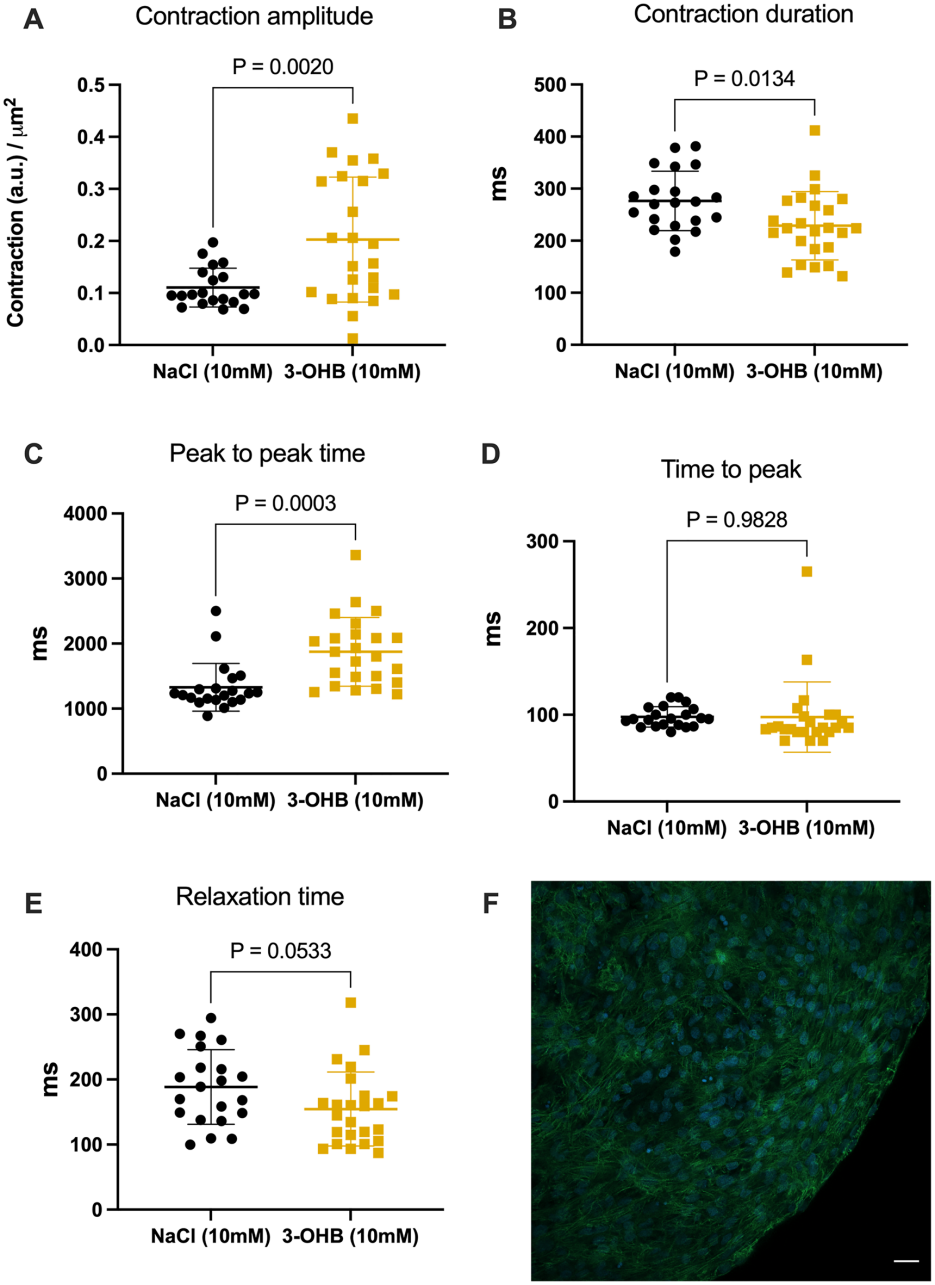
Micro organoid contraction patterns analysed by muscle motion 20 min after the addition of 10 mM NaCl or 3-OHB. (**A**) Concentration amplitude relative to cardiac organoid area. (**B**) Contraction duration. (**C**) Peak-to-peak time. (**D**) Time to peak. (**E**) Relaxation time. (**F**) Immunofluorescent staining demonstrating cardiac troponin I positive cells. Nuclear staining marker DAPI (blue) and CY2 cardiac troponin (green) are shown. Scale bar: 20 µM. *NaCl* sodium chloride, *3-OHB* sodium-3-hydroxybutyric acid. Data are mean with bars indicating standard deviation. P < 0.05 was considered significant.

Contraction amplitude was higher in 3-OHB-treated organoids than in control organoids (contraction a.u./organoid area mm^2^: 0.2 ± 0.12 vs. 0.11 ± 0.04, P = 0.002) (Fig. 4). The contraction duration was shorter in 3-OHB than in the control (ms: 229 ± 66 276 ± 57 ms, *P* = 0.013), whereas the peak-to-peak time (the signal cycle length) was higher in 3-OHB (ms: 1876 ± 528 vs. 1328 ± 368, *P* = 0.0002) (Fig. 4).

The relaxation time was shorter, although not statistically significant, in the 3-OHB group than in the control group (ms: 155 ± 57 vs. 189 ± 57, P = 0.053), whereas the time to peak was unchanged (3-OHB vs NaCl (ms): 97.5 ± 40.5 for vs. 97.7 ± 11.7, P = 0.982) (Fig. 4).

### Calcium imaging results

Calcium imaging showed no significant change in calcium handling properties between 3-OHB and the control in amplitude (3-OHB vs. control paced at 1 Hz (a.u.): 0.52 ± 0.22 vs. 0.43 ± 0.1, P = 0.54, rise time (3-OHB vs. control: paced at 1 Hz: 190.6 ± 28.1 ms 186.7 ± 118.4 ms, P = 0.957) or action potential duration (Fig. S4).

### Optical mapping results

Conduction velocity was not significantly modified by 3-OHB: (3-OHB vs. control paced at 1 Hz: 4.4 ± 1.2 cm/s vs. 5.3 ± 1.5 cm/s, P = 0.19) (Fig. S5).

## Discussion

We investigated the effects of 3-OHB on (1) hemodynamic and mitochondrial function assessed in vivo with PV loops and high-resolution respirometry following circulatory death with subsequent NRP and cardiac transplantation and (2) cardiac contractility, calcium handling and electrical properties in in vitro cardiac organoids.

First, we demonstrated that infusion with 3-OHB following circulatory death and NRP increased CO substantially while reducing arterial elastance compared with Ringer’s acetate infusion, without affecting mitochondrial function. Second, mitochondrial function was restored by 3-OHB after cardiac transplantation. Third, evidence of increased contractility was observed in vivo as 3-OHB increased dP/dt max compared with Ringer’s acetate. Last, we demonstrated that 3-OHB improved cardiac contractility in the hiPSC-based micro-organoid system without affecting the electrophysiological properties.

3-OHB has a beneficial effect in HF patients and healthy human volunteers by increasing CO up to 40%^13^, but it was speculated whether the increase was caused by vasodilation, increased contractility or a combination of the two. The present study demonstrated a marked reduction in arterial elastance by 3-OHB infusion, which most likely contributed significantly to the marked increase in CO and SV observed after NRP. However, a concomitant and significant increase in dP/dt max after NRP was observed and may indicate an improvement in contractility as well. These two cardiovascular targets of 3-OHB were recently documented in isolated perfused hearts and isolated arteries, showing independent inotropic and vasorelaxant effects^15^.

Our in vivo data support a direct effect of 3-OHB on contractility, but the improved functional recovery following circulatory death may also stem from ameliorated IR damage, as indicated by previous data reporting cardioprotective abilities by 3-OHB^9,10^.

As EDV and EDP remained constant throughout the experiment, we found no indication of an effect of 3-OHB on preload. MAP and HR were unaffected. Consequently, the Frank-Starling relationship shifted upwards to increase SV and CO due to a decrease in resistance, since arterial elastance is defined as the product of heart rate and resistance^34^. Our data suggest that 3-OHB administration in donor subjects has a beneficial effect on cardiac function that may improve cardiac outcome in the recipient and enable an increase in the number of donor hearts that qualify for transplantation^35^. However, our data also raise concerns due to extensive vasodilatation in the recipient animals that prevented weaning. While we aimed to achieve the highest effect by administering 3-OHB to both the donor and the recipient, our results indicate that this approach does not comply with a beneficial clinical strategy. The explanation for the extensive vasoplegia is unclear. Although posttransplant vasoplegia is expected^36^, weaning was successful in all donor pigs after NRP with 3-OHB. The hearts were transplanted to 3-OHB naïve bodies, so any carry-over of 3-OHB effects should not be present, and plasma concentrations of 3-OHB were similar before and after transplantation (Fig. S1). Our anticipation is that one of the mechanisms of 3-OHB acting as a potent vasodilator in combination with expected posttransplant vasoplegia challenges hemodynamic compensation. Both recipient groups received the potent inodilator milrinone as a bolus shortly before weaning. It may be speculated that this can have resulted in deleterious synergy with 3-OHB, causing excessive vasodilatation. Administration of 3-OHB to the donor only represents the next step to clarify its potential for cardioprotection in relation to DCD and NRP in human heart transplantation^5–7^

The purpose of NRP is to allow functional assessment of the heart before transplantation to optimize cardiac function in the recipient^4,35^. This is in contrast with transplantation with direct organ procurement. Our data underline the proposed advantages of NRP^4^, since we were able to assess and modulate cardiac function before transplantation. Future studies are needed to clarify whether this results in better cardiac performance on a larger time scale than that investigated here.

A key question relevant to the effect of 3-OHB is whether improved hemodynamics are mediated via a direct increase in cardiac contractility or whether it is related to an indirect effect through afterload reduction and vasodilatation. The results of the microorganoid tissue experiments show that 3-OHB directly increases the contractile surrogate for force and the contraction amplitude. In isolated perfused rat hearts mounted in Langendorff systems with constant pre- and afterload, 3-OHB increases cardiac contractility at 3–10 mM, which is in line with our findings in both organoids and pigs^15^. Therefore, we conclude that at least part of the improved hemodynamic effect of 3-OHB is mediated by improved contractility. In addition, we did not see any significant effect on conduction velocity or calcium handling upon exposure of micro-organoids to 3-OHB, and, consequently, the mechanism behind the increase in cardiac contractility did not involve changes in intracellular calcium.

In the present study, mitochondrial oxidative phosphorylation (OXPHOS) capacity related to both complexes I and II was clearly impaired by IR injury in both groups. 3-OHB increased CO and SW, but this did not affect mitochondrial OXPHOS capacity. Following SCS and reperfusion, OXPHOS capacity was salvaged by 3-OHB loading of the donor pig, while control pigs exhibited a continuous decline in OXPHOS capacity. Indeed, OXPHOS capacity measured by high-resolution respirometry is correlated with cardiac function in both terminal heart failure patients and heart transplant recipients^37,38^. From our data, 3-OHB preserves OXPHOS capacity following transplantation (Fig. 3). Even though our experimental setup differs from heart failure patients and heart transplant recipients, preservation of mitochondrial OXPHOS capacity has previously been associated with cardioprotection in animal models of IR damage^22,39–41^.

As an explorative option, we compared the respiratory control ratio with glucose-linked substrates in the high-resolution respirometry chamber with 3-OHB-linked substrates (Fig S2). It appears that IR and transplantation caused a shift in energy substrate metabolism towards metabolization of 3-OHB that increased mitochondrial respiration, independent of exposure to 3-OHB in the pigs during transplantation. This may support the hypothesis that ketones serve as alternative energy substrates during ischemic stress^42,43^. The cardiac adaptation towards metabolization of ketone bodies in HF has been extensively studied in recent years and is believed to be caused by regulation of key enzymes in ketone metabolism^42,44–46^. Our data suggest that the mitochondrial energy substrate shift in response to IR works within hours. Hence, it is unlikely explained by the increased abundance of katabolic enzymes alone. Especially within energy metabolism, other mechanisms, such as acetylation, are responsible for nonenzymatic regulation that might explain this rapid adaptation to energy substrate availability^42,47^. Further studies are needed to clarify the mechanism behind this energy substrate shift.

We did not investigate cardiac hemodynamic function following implantation because 3-OHB caused vasoplegia in recipients that did not allow weaning. The VIS score in donor pigs showed no significant differences between groups (Supplementary table S1). Unfortunately, reliable data for recipient pigs are unavailable due to the administration of multiple, undocumented boluses of inotropic drugs during unsuccessful weaning attempts. However, weaning with long-term survival was not the scope of this study. Further studies are needed to clarify whether administration of 3-OHB in donors alone offers similar effects as observed here.

The plasma sodium concentration increased following infusion with 3-OHB (Table S3). The potential clinical implications of this should be considered^48^. Hypertonic saline increases contractility following cardiac transplantation and may contribute to the increase we observed in dP/dt max^49,50^. However, from the in vitro study, we learned that iso-osmolar NaCl did not increase contraction amplitude, so the effect is unlikely explained by sodium alone. Therefore, comparison with control fluids that are balanced in osmolytes should be considered in future studies.

We exclusively used female pigs in this study, which may limit generalizability to both sexes. Nevertheless, our recent documentation of comparable hemodynamic and ex vivo responses to 3-OHB in healthy male rats and male humans suggest independence in the response of both species and sex^13,15^.

Permeabilized fibers to evaluate mitochondrial respiratory capacity is a validated method to investigate mitochondrial function^21–23,51^. Even so, the *in-situ* preparation is not a physiological milieu, so it remains a limitation whether our results translate on a whole organ level. Therefore, high resolution respirometry offers insights into mitochondrial function within a controlled extracellular environment but does not evaluate cellular energetics in intact tissue^52^. Respiratory results on human skeletal muscle using permeabilized fibers are in excellent agreement with data on isolated mitochondria in respirometry^53,54^. Nonetheless, the effect of 3-OHB on mitochondrial function should be further validated using physiological levels of ketones and in intact tissue and should be compared with oxidation of glucose and free fatty acids. With an increase in both mitochondrial OXPHOS capacity and state 4o leak respiration, it can be difficult to disentangle whether the increased membrane potential results in increased ATP production or whether it is channeled through the inner mitochondrial membrane and dissipate as thermic energy or through another pathway. Even so, comparison of the RCR and coupling efficiency does not guarantee correct interpretation^55^, so this aspect remains a limitation for the interpretation of our results.

Although within the same physiological range between 3 and 4 mmol/L, plasma concentrations of 3-OHB were higher in our study than in previous clinical studies^13,56^. Furthermore, we did not use the same target concentration of 3-OHB in vivo as we did in vitro, where the concentration was 10 mM. This was based on experience from other preclinical studies conducted in our research group and recently published^15,57^.

To conclude, 3-OHB administration had beneficial cardiac hemodynamic effects by increasing cardiac output and cardiac contractility and reducing arterial elastance following circulatory death and NRP. While 3-OHB seemed to maintain mitochondrial respiratory capacity after transplantation, administration of 3-OHB in the recipient pig caused inotropy-resistant vasoplegia that prevented weaning. Further studies are therefore needed to optimize administration practice in the donor and recipient setting and to validate the effect of 3-OHB on mitochondrial function in intact tissue.

### Supplementary Information

Supplementary Information.

## Data Availability

Original data underlying this article will be shared on reasonable request to the corresponding author.

## Abbreviations

3-OHB: 3-Hydroxybutyric acid
HiPSC: Human induced pluripotent stem cells
Ea: Arterial elastance
HR: Heart rate
CPB: Cardiopulmonary bypass
HF: Heart failure
CO: Cardiac output
IR: Ischaemia–reperfusion
DCD: Donation after circulatory death
MAP: Mean arterial pressure
EDV: End diastolic volume
NRP: Normothermic regional perfusion
EDP: End diastolic pressure
SCS: Static cold storage
ESV: End systolic volume
SV: Stroke volume
ESP: End systolic pressure
