# Supplementary material for: Effects of ketone body 3-hydroxybutyrate on cardiac and mitochondrial function during donation after circulatory death heart transplantation
Source: Sci Rep. 2024 Jan 8;14:757. doi: 10.1038/s41598-024-51387-y (PMC10774377; doi:10.1038/s41598-024-51387-y)
Supplement: Supplementary file 1 — Supplementary Information. [file 41598_2024_51387_MOESM1_ESM.pdf]

Effects of ketone body 3-hydroxybutyrate on cardiac and mitochondrial function during donation after circulatory death heart transplantation

Supplementary materials, methods, and results

Authors:
*⁰Jacob Marthinsen Seefeldt[1,2], M.D. (https://orcid.org/0000-0002-0633-5878)
⁰Yaara Libai, BSc.[3] (https://orcid.org/0009-0001-0862-6775)
Katrine Berg[1,2], M.D. (http://orcid.org/0000-0001-9309-4701)
Nichlas Riise Jespersen[1], MD., PhD., (http://orcid.org/0000-0003-2502-2102)
Thomas Ravn Lassen[1], MD., PhD., (https://orcid.org/0000-0003-4765-060)
Frederik Flyvholm Dalsgaard[1,4], bsc.med. (http://orcid.org/0000-0002-7287-9793)
Pia Ryhammer[5], MD (http://orcid.org/0000-0002-9290-8205)
Michael Pedersen[4], Prof., PhD (http://orcid.org/0000-0002-1146-0382)
Lars Bo Ilkjaer[6], MD (http://orcid.org/0000-0002-2340-1275)
Michiel A. Hu[7], MD. (https://orcid.org/0000-0002-7355-099X)
Michiel E Erasmus[7], MD. PhD. (https://orcid.org/0000-0002-6234-2976)
Roni R Nielsen[1], MD., PhD (https://orcid.org/0000-0001-9228-3869)
Hans Erik Bøtker[2], Prof., MD., PhD., DMSc, (https://orcid.org/0000-0001-6358-8962)
#Oren Caspi, MD, PhD[3] (https://orcid.org/0000-0002-2464-7357)
#Hans Eiskjær[1], Prof., MD., PhD., DMsc., (https://orcid.org/0000-0003-1520-1924)
#Niels Moeslund[6], MD., PhD. (https://orcid.org/0000-0003-0827-5263)

[1] Department of Cardiology, Aarhus University Hospital, Palle Juul-Jensens Boulevard 99, 8200 Aarhus N, Denmark.
[2] Department of Clinical Medicine, Aarhus University, Palle Juul-Jensens Boulevard 82, 8200 Aarhus N, Denmark.
[3] The laboratory for Cardiovascular Precision Medicine, Rapport Faculty of Medicine, Technion and Rambam's Cardiovascular Research and Innovation Center, 2 Efron St, Haifa, Israel.
[4] Comparative Medicine Lab, Department of Clinical Medicine, Aarhus University, Palle Juul-Jensens Boulevard 82, 8200 Aarhus N, Denmark.
[5] Department of Anesthesiology, Region Hospital Silkeborg, Falkevej 1A, 8600 Silkeborg, Denmark
[6] Department of Cardiothoracic and Vascular Surgery, Aarhus University Hospital, Palle Juul-Jensens Boulevard 99, 8200 Aarhus N, Denmark.
[7] Department of Cardiothoracic Surgery, University Medical Center Groningen, Hanzeplein 1, 9713 GZ Groningen, The Netherlands
⁰Equal Contribution.
#The authors share last authorship.

*Corresponding author:
Jacob Marthinsen Seefeldt
Telephone: +45 41 17 86 33
Email: Jacob.Seefeldt@clin.au.dk
Address: Falstersgade 53, 2., 8000 Aarhus C, Denmark

Table of content

SUPPLEMENTARY METHODS 2

ANESTHESIA, MONITORING, AND BASELINE MEASUREMENTS 2
DONOR PROCEDURE 2
DCD, NRP AND INTERVENTION 2
RECIPIENT PROCEDURE 3
MITOCHONDRIAL RESPIRATORY CAPACITY 4
IN VITRO HIPSC STUDY 5
MICRO-ORGANOID TISSUE ENGINEERING 6
MICRO-ORGANOID TISSUE ANALYSIS 6
CALCIUM IMAGING 7
OPTICAL MAPPING 7

SUPPLEMENTARY RESULTS 8

PROTOCOL FEASIBILITY 8
HEMODYNAMIC EVALUATION 10
PLASMA CONCENTRATION OF 3-OHB 12
MITOCHONDRIAL RESPIRATORY CAPACITY 13
MICRO ORGANOID TISSUE BASELINE VALUES 14
CALCIUM IMAGING 15
OPTICAL MAPPING 16

REFERENCES 17

Supplementary methods

Anesthesia, monitoring, and baseline measurements

Premedication was Zoletil Vet 50, (ketamine (6.25 mg/mL), tiletamine (6.25 mg/mL), benzodiazepine (6.25 mg/mL), synthetic opioid (butorphanol) (1.25 mg/mL), and xylazine (6.5 mg/mL)). Animals were mechanically ventilated with a tidal volume of 8 mL/kg, FiO2 of 40%, PEEP of 5 cm H_2O and respiratory frequency of 12-15 to maintain an end-tidal CO_2 of 4.5-5.6 kPa. Anesthesia and analgesia were maintained with inhaled Sevoflurane (3%) and fentanyl (15 µg/kg/h). Infusion of amiodarone (10 µg/kg/min) and a bolus of 100 mg lidocaine were administered to stabilize heart rhythm during the experiment. Both donor and recipient received 1.5 g Cefuroxime and 1000 mg Solu-Medrol at the start of the procedure. Plasma concentrations of 3-OHB were measured using a point of care device (Freestyle Precision Neo, Abbott, USA).

Donor procedure

Premedication was administered at the housing facility before transport to the surgical facilities. Via the right femoral artery, an aortic occlusion balloon was advanced to the abdominal aorta above the iliac bifurcation. After sternotomy, umbilical tapes were placed around the aortic arch vessels and inferior vena cava. A Pressure-volume catheter was inserted in the left ventricle via the left carotid artery. The standard roller pump CPB (cardiopulmonary bypass) circuit was primed with 1000 mL Ringers lactate, 200 mL mannitol and 100 mL 8.4% sodium bicarbonate.

DCD, NRP and Intervention

Functional warm ischemic time (fWIT) was defined as the time from systolic blood pressure <50 mmHg to the onset of NRP. Circulatory arrest was defined as central venous pressure (CVP) = mean

arterial pressure (MAP). Three minutes before NRP, the abdominal aortic balloon was inflated and pulled distally to the iliac bifurcation and aortic arch vessels ligated. NRP was commenced for 60 min, and the ventilation was restarted with a FiO2 60% and titrated to PaO_2 levels between 12–16 kPa. Norepinephrine was used to maintain MAP >60 mmHg during NRP. At 5, 15 and 55 min after onset of NRP, arterial and mixed venous blood samples were collected. Biochemical parameters including arterial pH, K^+, and glucose were corrected during NRP. Fifteen min before weaning from NRP, infusion of dobutamine (2.5 μkg/kg/min) was started and the heart gradually loaded and weaned from NRP.

Recipient procedure

Anesthesia for the recipient animals were maintained with propofol (3.5 mg/kg/h) and fentanyl (15 μg/kg/h). Baseline hemodynamic measurements were performed before start of infusion of 3-OHB or placebo for the duration of the experiment. Systemic anticoagulation with 40.000 IU heparin and ascending aortic and bi-caval cannulation were used to institute CPB. Flow rates were adjusted to MAP > 60 mmHg and normothermia was maintained throughout the recipient procedure.

The order of anastomosis was left atrium, inferior vena cava, superior vena cava followed by 250 mL antegrade HTK cardioplegia before pulmonary artery and finally aorta were anastomosed. A vent was placed in the apex of the LV and de-airing maneuvers were performed before removal of the aortic cross-clamp and reperfusion of the donor heart. Internal defibrillation (30 J) was used to treat ventricular arrhythmias. Dobutamine (1.5 – 2.5 μg/kg/min) and norepinephrine (0.05 – 1.0 μg/kg/min), were used for inotropic and hemodynamic support, respectively. The hearts were gradually volume-loaded and CPB flow decreased; weaning was considered successful if the animals maintained a MAP above 50 mmHg for 30 min after discontinuation of CPB.

Mitochondrial respiratory capacity

Biopsies were immediately transferred to a biopsy preserving solution (BIOPS: 10 Ca-EGTA buffer, 10^{-4} free Ca^{2+}, 20 imidazole, 20 taurine, 50 K-MES, 0.5 dithiothreitol, 6.56 $MgCl_2$, 5.77 ATP, and 15 phosphocreatine, pH 7.1). Fiber bundles (~1.5mm) were prepared by manual dissection and permeabilized in cold BIOPS solution mixed with saponin (50 µg mL^{-1}) by gentle agitation for 30 min. Hereafter, fibers were rinsed twice in cold respiration medium, MiR05 (in mmol L^{-1}: 110 sucrose, 60 K-lactobionate, 0.5 EGTA, 0,1% BSA, 3 $mgCl_2$, 20 taurine, 10 KH_2PO_4 and 20 Hepes; pH 7.1).

The permeabilized fiber bundles were added to the chambers in the Oxygraph-2k, filled with 2mL MiR05 at 37°C.

To evaluate physiological mitochondrial respiration, the following substrate-uncoupler-inhibitor-titration (SUIT) protocol was used: Glutamate (10 mmol/L) and Malate (2 mmol/L) without presence of ADP stimulate basal complex I respiration. Subsequent addition of ADP (5 mmol/L) allows complex I mediated respiration with electron flow through the mitochondrial ATPase (GM3). Succinate (10 mmol/L) was added to stimulate maximal respiration with electron flow through complex I+II (GMS3). Oligomycin (complex V inhibitor) (2 µg/mL) was added to evaluate state 4o leak respiration. Final addition of rotenone (complex I inhibitor) (0.5 µmol/L) and antimycin A (complex III inhibitor) (2.5 mmol/l) allows measurement of residual oxygen consumption.

To evaluate respiration driven by oxidative phosphorylation of ß-hydroxybutyric acid, the following SUIT protocol was used: Glutamate (10 mmol/L) and Sodium-3-OHB (in titration steps to reach maximal respiration) stimulates basal mitochondrial respiration without ADP. Subsequent addition of ADP (5 mmol/L) allows evaluation of respiration through complex I. Final

addition of succinate (10 mmol/L) stimulates maximally coupled respiration with electron flow through complex I+II.

To avoid any O_2 limitations to respiration the chambers were hyperoxygenated and all measurements were carried out in duplicate. The integrity of the outer mitochondrial membrane was tested by the addition of cytochrome c (10 μmol/L). An increase of >10% compared with complex I mediated coupled respiration (GM3) in the oxygen consumption rate led to exclusion.
The data were corrected for residual oxygen consumption and the OXPHOS capacity was calculated as respiration for complex I+II subtracted with leak state respiration[1]. We calculated the respiratory control ratio (RCR) as maximal respiration (GMS3 for glucose linked substrates or GBS3 for 3-OHB substrates) divided by leak state (GM or GOHB).
The RCR was calculated as maximally coupled O_2 flux (GMS3) / baseline O_2 flux (GM).

In vitro hiPSC study

Human fibroblasts were reprogrammed by retroviral delivery of three reprogramming factors to obtain the patient-specific human induced pluripotent stem cells (hiPSCs) as previously described (SOX2, KLF4 and OCT4)[2,3]. Undifferentiated hiPSC were grown in mTeSR Plus (Stemcell technologies, Vancouver, Canada) on Matrigel coated plates and passaged every 4 days at 1:10 ratio using 5mM EDTA solution (Life Technologies, California, USA).
The cells were differentiated into cardiomyocytes by applying CDM3 with 6µM CHIR99021 (Tocris, Bristol, UK) to cell confluency of 85%. After 2 days, the medium was changed to CDM3 with 2µM Wnt-C59 (Selleck Chemicals, USA). 2 days later, the medium was changed to RPMI-1640 (Gibco Dynamics Medium, Thermo Fisher, Massachusetts, USA) with B27 (Thermo fisher, Massachusetts, USA) without insulin,based on a previouslyestablished cardiomyocyte differentiation protocol[4].

Micro-Organoid Tissue engineering

Human iPSCs derived cardiomyocytes grown as monolayers were detached using TrypLE express X1 for 5 minutes at 37C^{0}. The detached cardiomyocytes were seeded on a V-bottom 96 well plate (Thermo Fisher, Massachusetts, USA) in a concentration of 10^4 cells/50μL for each well. Microplates were then centrifuged for 10 min at 1100 rpm. Micro-organoids were incubated at 37C^{0}, 5% CO2 for 7 days with media (RPMI-B27 without insulin) refreshed every 2-3 days. After 7 days, video recordings were carried out using a robotic inverted microscope (Olympus IX83, Olympus, USA). The organoids were video recorded for 8 seconds (for each well) in a Okolab Incubator (Italy) at 37C^{0} and in the presence of 5% CO_2.

Micro-Organoid Tissue Analysis

All signals shorter than 200ms and longer than 600ms were excluded. Outliers were calculated using a Python script that calculates the quantiles, IQR, and the upper and lower limitation. The outlier signals were excluded from the calculation of the average of the micro-organoid signal. Bazett's formula was used for calculating the contraction duration and relaxation time (calibrating for the variability in the beating rate). The total number of wells that were included in the analysis are 24 wells of 3-OHB and 21 wells of NaCl.

Calcium Imaging

HiPSC were plated in 35mm glass-bottom dish with 10mm micro-well (Cellvis, California, USA) in a concentration of 10^5 cells/140µL. Media (RPMI-B27 without insulin) was refreshed every 2 days. After 7-10 days, the cells were loaded with the calcium indicator Fluo-4 AM (Thermo Fisher, Massachusetts, USA). Cells were incubated with 1µL Fluo-4/1mL of RPMI-B27 for 30 minutes and were measured in Tyrode's solution (140 mM NaCl, 5.4 mM KCl, 1.8 mM CaCl2, 1 mM MgCl2, 5 mM HEPES, 10 mM glucose; pH 7.4). Fluorescent signal changes were acquired in a line-scan mode for optical-signal analysis for depicting changes in cell-fluorescence. Four wells of each group (control and 3-OHB) were included in the analysis.

Optical Mapping

IPS derived cardiomyocytes were plated in surface 35mm culture dish (Corning CellBIND, New York, USA) in a monolayer form, in the concentration of 1 million cells/50µL. Media was refreshed every two days with 2mL RPMI-B27 without insulin. Blebbistatin (5 µM, Sigma-Aldrich, Missouri, USA, B0560) was added to the media in a 1:2000 ratio, to facilitate monolayer condensation and to prevent from formation of micro-tears creating holes within the monolayer. After 7-10 days, the cells were loaded with FluoVolt Membrane Potential dye Kit (F10488, Thermo Fisher, Massachusetts, USA) for 30 minutes. 2D cardiac tissues were then maintained in a heated organ bath at 37°C in standard Tyrode's solution (140 mM NaCl, 5.4 mM KCl, 1.8 mM CaCl2, 1 mM MgCl2, 5 mM HEPES, 10 mM glucose; pH 7.4). A custom-made setup was used to observe fluorescence changes which were directly proportional to transmembrane voltage changes. Blue LED illumination was used, and spontaneous or electrically excited action potentials were

recorded with the CCD camera via appropriate emission filter (515 nm, green light, Chroma). The default field of view (FOV) achieved for illumination and imaging by using the 1X objective and setting the zoom body on 2.5X was 10mm. Fluorescence was acquired at 4x4 binning, and a sampling interval of 3.847ms (~260 frames per second) at all experiments. Calculation of conduction velocity and action potential duration was performed using the optical and electrophysical mapping software ElectroMap[5]. Five wells paced at 1Hz were included in the analysis.

Supplementary results

Protocol feasibility

Donor times and VIS score (Median (IQR))			
	Control N=9	3-OHB N=5	P-value (Mann-Whitney test)
W⇒SBP<50 mmHg (min)	2 (1 – 5)	1 (1 – 2)	0.23
SBP<50 mmHg⇒CA (min)	3 (1.5 – 4.5)	3 (1.5 – 7)	0.54
W ⇒ CA (min)	6 (4 – 9)	4 (3 – 8.5)	0.62
fWIT(SBP<50⇒NRP) (min)	13 (11.5 – 14.5)	13(11.5 – 17)	0.54
VIS-score	1.3 (0.68 – 4.7)	2.5 (1.35 – 3.36)	0.90

Supplementary table 1. First row is the average time from W: withdrawal from life support until SBP: systolic blood pressure less than 50mmHg. Second row is the average time from SBP less than 50mmHg undtil CA: circulatory arrest. Third row is the average time from W to CA. Fourth row is the average time from SBP less than 50mmHg until NRP: normothermic regional perfusion, also named fWIT: functional warm ischemic time. Fifth row is the vasoactive inotropic score (VIS).

Transplantation times (Median (IQR))			
	Control<br>N=9	3-OHB<br>N=5	P-value (Mann-Whitney test)
Static cold storage (min)	125 (115 – 135)	135 (99 – 139)	0.78
Implant time (min)	101 (84 -116)	105 (100 – 130)	0.37
Total ischemia (static storage + implantation) (min)	224 (212 – 244)	240 (231 – 246)	0.25
CPB reperfusion (min)	111 (85 – 146)	108 (103 – 113)	0.70

Supplementary table 2. CPB: Cardiopulmonary Bypass

Hemodynamic evaluation

Variable	Baseline		Post-NRP		P-value<br>Baseline<br>3-OHB vs.<br>Placebo	P-value<br>Post-NRP<br>3-OHB vs.<br>Placebo
	3-OHB<br>N=5	Placebo<br>N=9	3-OHB<br>N=5	Placebo<br>N=9		
P-3-OHB (mmol/l)	0.1±0.1	---	4.1±1.0*	---	---	---
MAP (mmHg)	79±8	70±14	105±18*	93±23*	0.4	0.3
CO (L/min)	6.0±1.7	5.2±1	11.0±0.4*	5.8±2.0	0.7	<0.0001
SV (mL)	80±11	70±19	88±12	51±17	0.5	0.001
ESV (mL)	92±23	75±24	99±8	92±47	0.7	>0.99
EDV (mL)	168±30	132±55	184±50	138±64	0.5	0.3
EF (%)	49±10	50±7	49±4	39±15*	>0.99	0.2
Ea (mmHg/mL)	1.2±0.2	1.4±0.4	1.4±0.2	2.3±0.6*	0.9	0.003
Ees (mmHg/mL)	0.8±0.4	1.2±0.5	1.4±0.5	1.4±0.6	0.3	>0.99
dP/dt max (mmHg/s)	1454±510	1323±236	3598±1621*	2437±728*	>0.99	0.04
Heart rate (min^{-1})	74±11	78±16	129±23*	113±13*	>0.99	0.2
ESP (mm Hg)	92±6	87±12	120±22	112±33*	>0.99	>0.99

EDP (mm Hg)	12±6	11±5	11±5	13±8	>0.99	0.9
Stroke Work (mmHg*mL)	7091±1940	5631±1692	10992±3779	5665±2435	0.6	0.001
SVR	13±2	14±2	17±8	19±8	>0.99	>0.99
pH	7.50±0.14	7.49±0.05	7.39±0.01	7.41±0.07	0.6	>0.99
Lactate	1.2±0.3	1.8±0.7	8.0±1.1*	6.9±1.5*	0.6	0.2
Na^+	139±1	139±1	148±2*	140±1	0.6	<0.0001
Glucose	6.1±1.4	6.0±2.4	5.5±2.6	5.9±1.1	0.9	0.8

Supplementary table 3. Hemodynamic recordings from PV loops. *P<0.05, **P<0.01, ***P<0.001, ****P<0.0001, compared with baseline.

Plasma concentration of 3-OHB

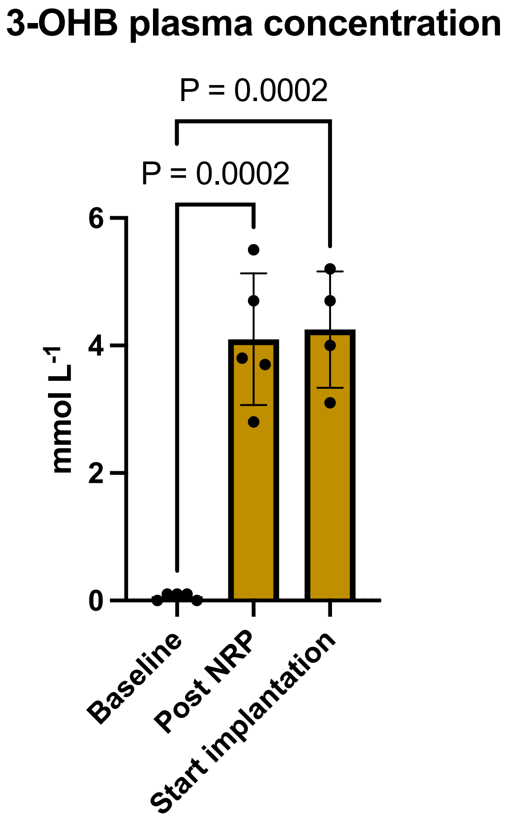

Supplementary figure 1. Plasma concentrations of 3-OHB during the experimental protocol.
NRP: Normothermic Regional Perfusion.
Data are means with bars indicating standard deviation. P<0.05 considered significant.

Mitochondrial respiratory capacity

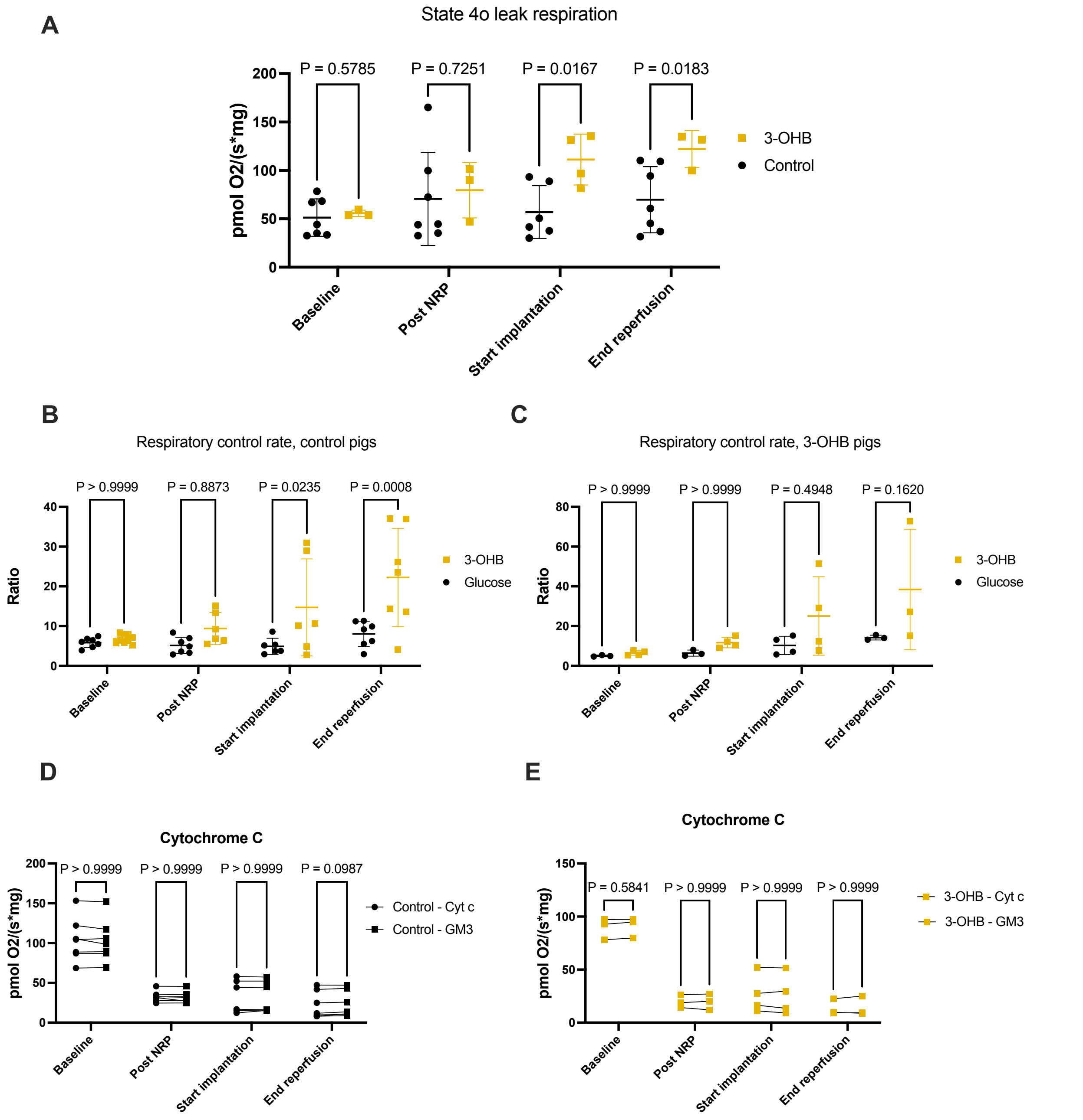

Supplementary figure 2. Mitochondrial respiration at different respiratory states. A: State 4o leak respiration with oligomycin B: Respiratory control ratio of control pigs with 3-OHB or glucose substrates in the oxygraphy chamber. C: Respiratory control ratio of 3-OHB pigs with 3-OHB or glucose substrates in the oxygraphy chamber. D-E: Increase in oxygen consumption from addition of Cytochrome c after GM3. *Data are mean with bars indicating standard deviation. P<0.05 considered significant.*

Micro organoid tissue baseline values

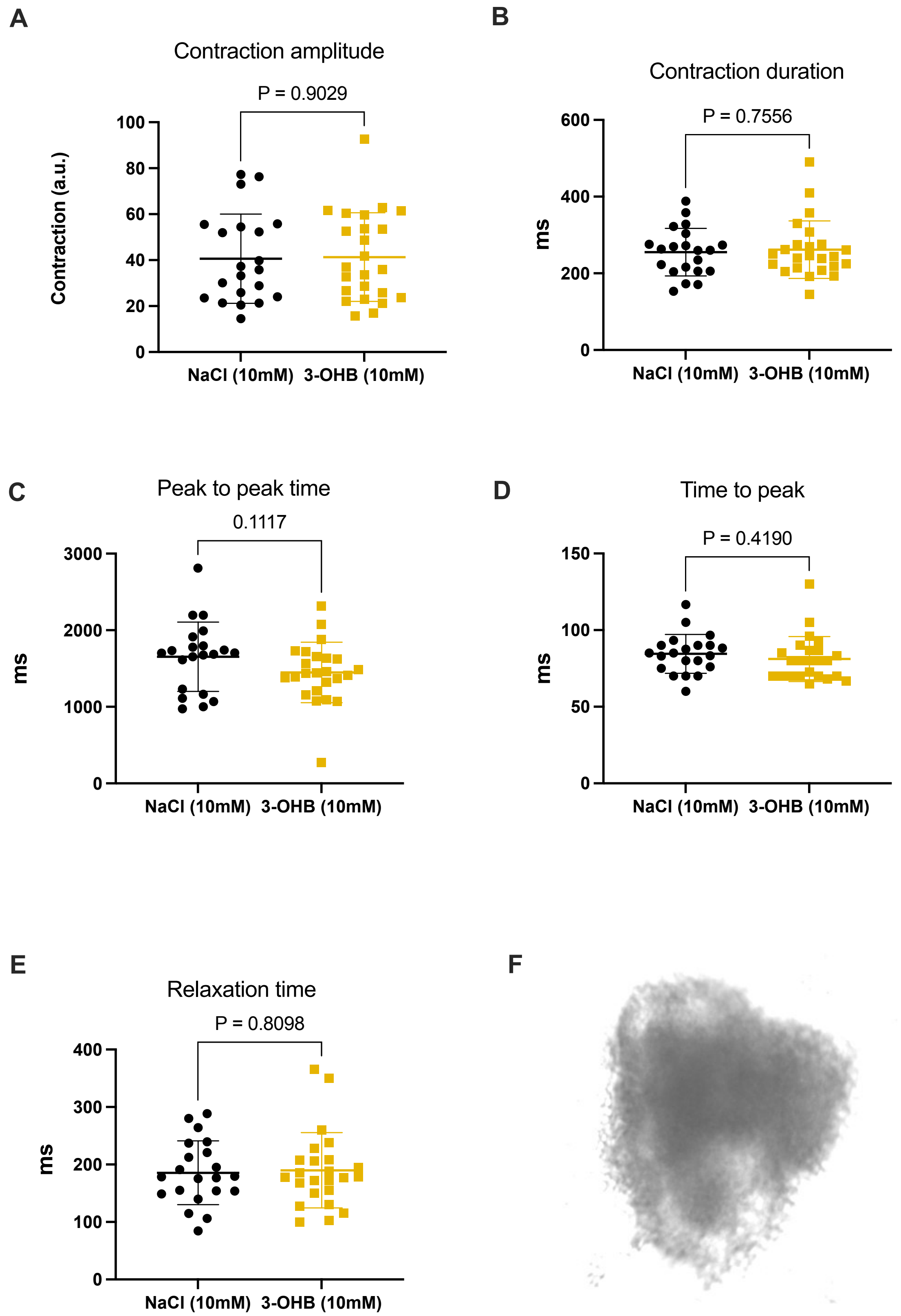

Supplementary figure 3. Micro organoids contraction patterns analyzed by MUSCLEMOTION analysis at baseline. A: contraction amplitude. B: contraction duration. C: Peak to peak time. D: Time to peak. E: Relaxation time. F: Micro organoid tissue as seen on video camera. *NaCl: Sodium-chloride. 3-OHB: sodium-3-hydroxybutyric acid. Data are mean with bars indicating standard deviation. P<0.05 considered significant.*

Calcium imaging

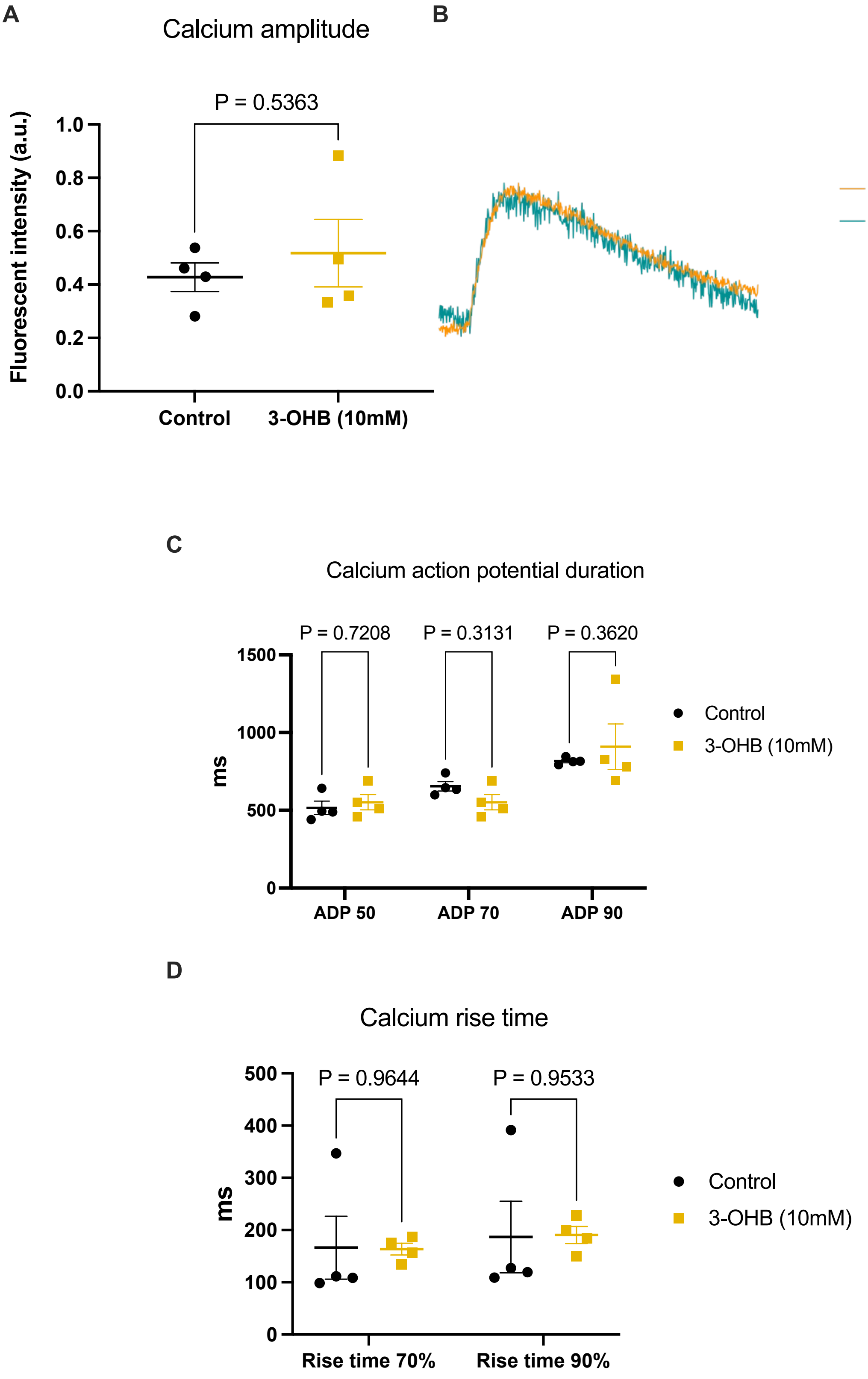

Supplementary figure 4. Calcium imaging (pacing at 1Hz) with calcium sensitive fluorescence of hIPSC after exposure to 3-OHB. A: Amplitude of fluorescence signal. B: Representative trace of fluorescence signal rise. C: Calcium action potential duration D: Calcium rise time. *3-OHB: sodium-3-hydroxybutyric acid. Data are mean with bars indicating standard error of mean. P<0.05 considered significant.*

Optical mapping

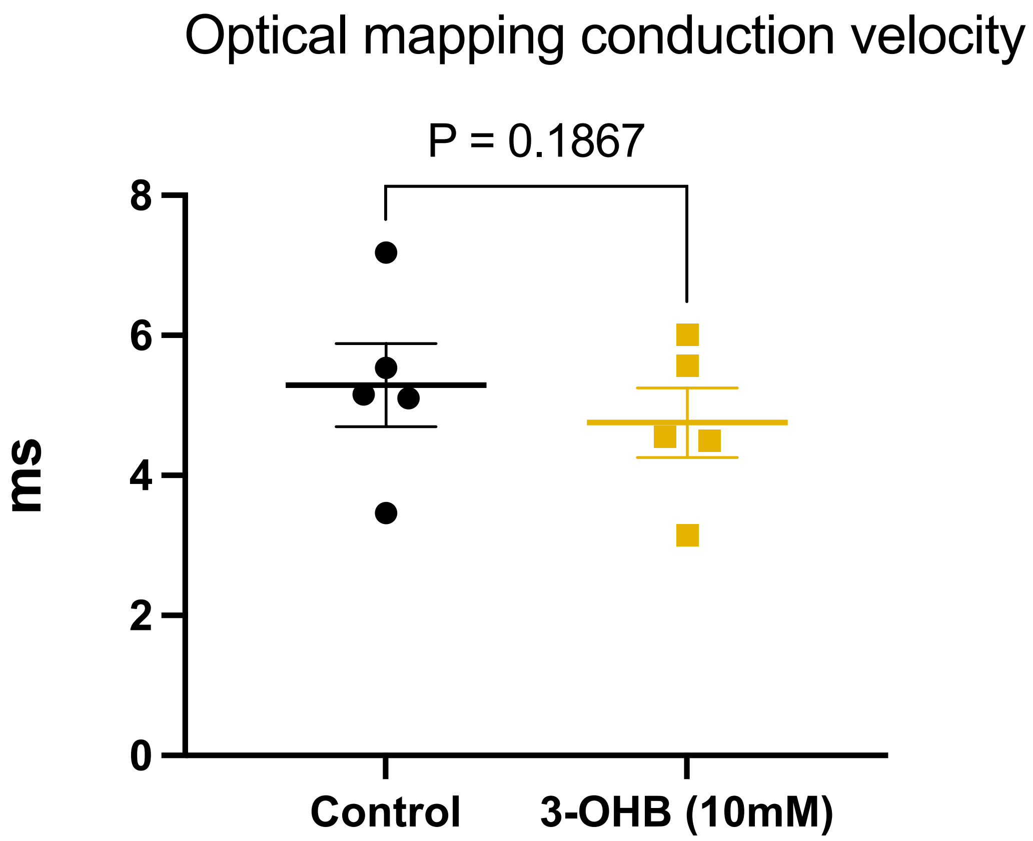

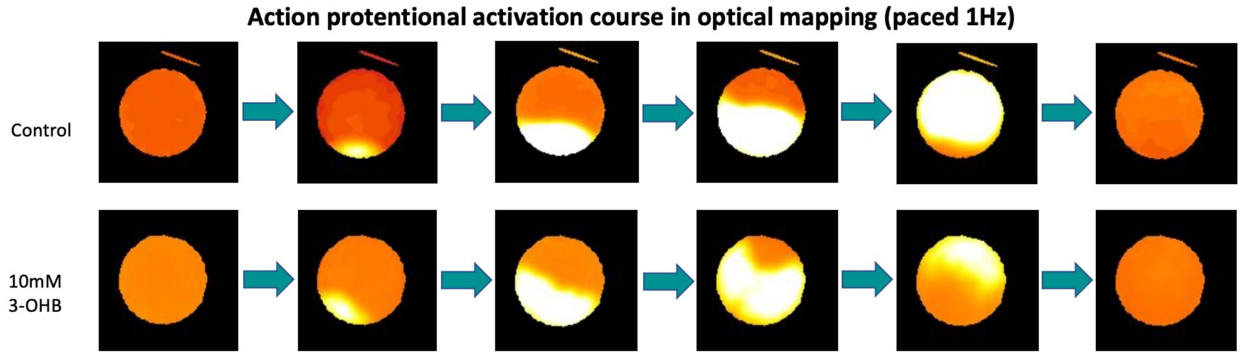

Supplementary figure 5. Optical mapping of conduction velocity (at 1Hz pacing) in hIPSC by use of voltage sensitive luminescence. A: Conduction velocity. B: Representative images in control and 3-OHB exposed tissue *3-OHB: sodium-3-hydroxybutyric acid. Data are mean with bars indicating standard error of mean. P<0.05 considered significant.*

References

1. Gnaicher E. Mitochondrial Pathways and Respiratory Control - an Introduction to OXPHOS Analysis. 2020;doi:10.1007/978-3-319-43287-8_7
2. Itzhaki I, Maizels L, Huber I, et al. Modelling the long QT syndrome with induced pluripotent stem cells. *Nature*. Mar 10 2011;471(7337):225-9. doi:10.1038/nature09747
3. Caspi O, Huber I, Gepstein A, et al. Modeling of arrhythmogenic right ventricular cardiomyopathy with human induced pluripotent stem cells. *Circ Cardiovasc Genet*. Dec 2013;6(6):557-68. doi:10.1161/CIRCGENETICS.113.000188
4. Lian X, Hsiao C, Wilson G, et al. Robust cardiomyocyte differentiation from human pluripotent stem cells via temporal modulation of canonical Wnt signaling. *Proc Natl Acad Sci U S A*. Jul 3 2012;109(27):E1848-57. doi:10.1073/pnas.1200250109
5. O'Shea C, Holmes AP, Yu TY, et al. ElectroMap: High-throughput open-source software for analysis and mapping of cardiac electrophysiology. *Sci Rep*. Feb 4 2019;9(1):1389. doi:10.1038/s41598-018-38263-2
